# Occupational Exposure to Blood and Body Fluids Among Health Care Workers in a Teaching Hospital in Mumbai, India

**DOI:** 10.4103/0970-0218.39239

**Published:** 2008-01

**Authors:** Samir A Singru, Amitav Banerjee

**Affiliations:** Department of Community Medicine, Dr. D.Y. Patil Medical College, Pune - 411 018, Maharashtra, India

**Keywords:** Health care workers, needle-stick injury, occupational exposures

## Abstract

**Objective:**

Exposure to blood and body fluids is one of the hidden hazards faced by health care workers (HCWs). The objective of the present study was to estimate the incidence of such exposure in a teaching hospital.

**Materials and Methods:**

A cross-sectional study among a random sample of residents, interns, nurses and technicians (*n* = 830) was carried out in a teaching hospital to estimate the incidence of exposure to blood and body fluids in the preceding 12-month period. Self-reported occurrence and the circumstances of the same were recorded by face-to-face interviews using a semi-structured questionnaire.

**Results:**

The response rate to the study was 89.76%. Occupational exposure to blood and body fluids in the preceding 12 months was reported by 32.75% of the respondents. The self-reported incidence was the highest among the nurses. Needle-stick injury was the most common mode of such exposures (92.21% of total exposures). Index finger and thumb were the commonest sites of exposure. Only 50% of the affected individuals reported the occurrence to concerned hospital authorities. Less than a quarter of the exposed persons underwent post-exposure prophylaxis (PEP) against HIV, although the same was indicated in about 50% of the affected HCWs based on the HIV status of the source patient.

**Conclusions:**

Occupational exposure to blood and body fluids was a common occurrence in the study sample. There was gross under-reporting of such incidents leading to a lack of proper PEP against HIV in 50% of those in whom the same appeared to be indicated.

## Introduction

Needle-stick injuries and cuts are the common occupational accidents exposing health care workers (HCWs) to blood and body fluids. These preventable injuries expose workers to over 20 different blood-borne pathogens(1) and result in an estimated 1000 infections per year, the most common being Hepatitis B, Hepatitis C and HIV.(2) According to World Health Report 2002, 2.5% of HIV cases among HCWs and 40% of Hepatitis B and C cases among HCWs worldwide are the result of occupational exposure.(3) Unlike developed countries, most developing countries may not have surveillance for occupational exposure to blood and body fluids, which precludes estimation of the exact magnitude of such accidents.

The present study was carried out to estimate incidence during the preceding 12 months of blood and body fluid exposures among HCWs in a teaching hospital, circumstances leading to such accidents and post-exposure actions taken by the HCWs.

## Materials and Methods

The study was carried out in a tertiary care teaching hospital in Mumbai, India. The metro is situated in the western coast of India and is the economic capital of the country, highly industrialized, with a comparative higher incidence of HIV infection as compared to the rest of the country. Written permission for conducting the study was taken from the hospital administrative authorities.

The HCWs included the following categories:

- Resident doctors: 450
- Interns: 300
- Staff nurses: 755
- Medical technicians: 45
- Total: 1550

Out of the above HCWs, 250 resident doctors, 200 interns, 350 staff nurses and 30 medical technicians (total 830) were selected for the study by stratified random sampling. After explaining the purpose of the study, consent for participation was taken from each HCW.

### Definition of occupational exposure

Accidental needle-stick injury was defined as a prick with a needle or other sharp object during use of the object for patient care. Accidental splash was defined as a splash of any body fluid from a patient onto the skin or mucous membrane of a HCW.

### Measurement of occupational exposure

Self-reported occupational exposure to blood/body fluids was elicited for the past one year from each subject using a semi-structured study instrument, which was pre-tested in a pilot study and suitably modified. Data from the pilot study were not included in the main study. The HCW was asked to recall exposure to blood and body fluids in the preceding 12-month period. They were also queried about the type of accident, circumstances leading to the exposure and the body site of exposure. Information was also elicited on what they did after encountering such exposures regarding local toilet, notification, lab investigation and post-exposure prophylaxis (PEP). The responses to the questionnaire were collected from the subjects by face-to-face interviews by trained interviewers.

## Results

### Response rate

Out of the 830 selected HCWs, 745 (238 resident doctors, 158 interns, 323 staff nurses and 26 medical technicians) agreed to participate in the study giving, a response rate of 89.76%.

### Incidence of occupational exposure to blood and body fluids

The overall incidence of occupational exposure to blood and body fluids during the study period of one year was 32.75%. The incidence of accidental exposure to potential infectious material was the highest among the staff nurses at 39.63%, followed by interns at 37.34%, technicians at 26.92% and least among the resident doctors at 21.01%.

### Type of accident leading to occupational exposure

This is shown in Table 1. Most of the exposures (92.21%) were due to needle-stick injuries. The rest (7.79%) were due to splashing of body fluids/blood.

**Table 1:** Type of accidents leading to occupational exposure

Category of HCW	Needle-stick injury body	Splashing of fluid/blood	Total
Residents	43 (86)	7(14)	50(100)
Interns	56 (94.92)	3 (5.08)	59(100)
Staff nurses	119(92.97)	9 (7.03)	128(100)
Technicians	7(100)	Nil	7(100)
Total	225(92.21)	19(7.79)	244 (100)

HCW - Health care worker, Figures in parentheses are in percentage

### Procedure-wise distribution of exposure to blood and body fluids

Overall, re-capping of needles was the most hazardous procedure particularly among interns and staff nurses. Drawing blood samples, setting up IV lines and giving injections were the other hazardous procedures exposing the HCWs to potential infectious material in order of frequency. Among resident doctors, surgical operations and conduct of labour were the common circumstances leading to exposure to blood and body fluids.

### Sites of exposure

The most common site of exposure was the non-dominant index finger (61.06%), followed by the non-dominant thumb (31.15%). Other less frequent sites were forearms (5.75%), mucosa/conjunctiva (1.23%) and legs (0.82%).

#### Washing of exposure site:

Table 2 shows the category-wise practice of washing the exposure site with soap and water. Greater proportion of nurses observed the desirable practice of washing the site with soap and water (82.03%), as compared to interns and residents.

**Table 2:** Washing of exposure site with soap and water

Category of HCW	Washed site with soap and water (%)	Total(%)
Residents	33 (66)	50 (100)
Interns	34 (57.63)	59 (100)
Nurses	105(82.03)	128(100)
Technicians	4 (57.14)	7 (100)
Total	176(72.13)	244 (100)

Nurses and technicians were clubbed for the analysis, χ^2^ = 12.09 df = 2 *P* = 0.002

#### Notification:

A much larger proportion of residents and interns (76% and 77.97%, respectively) notified the occurrence of occupational exposure as compared to only 26% of nurses reporting the incidence to the concerned hospital authority. This difference was statistically significant [Table 3].

**Table 3:** Notification to concerned authority after accidental exposure

Category of HCW	Notified (%)	Total (%)
Residents	38 (76)	50 (100)
Intems	46 (77.97)	59 (100)
Nurses	34 (26.56)	128(100)
Technicians	4 (57.14)	7(100)
Total	122(50)	244 (100)

Nurses and technicians were clubbed for the analysis, χ^2^ = 57.76, df = 2, *P =*0.0000, HCW-Health care worker

#### Exposure status of source patient:

The source patient was HIV negative in 52.87% of the occupational exposures; in only 6.97% of the exposures, the source patient was HIV positive; in the rest (40.16%), the HIV status of the source patient was unknown.

#### Proportion of HCWs undergoing lab investigations category-wise:

This is shown in Table 4. Significantly higher proportion of residents and interns underwent lab investigations as compared to nursing staff. Overall, only 36.48% of the HCWs underwent lab investigations after occupational exposure to blood and body fluids.

**Table 4:** HCW undergoing lab investigation after accidental exposure

Category of HCW	Underwent lab investigation	Did not undergo lab	investigation Total
Residents	26 (52)	24 (48)	50(100)
Interns	34 (57.63)	25 (42.37)	59(100)
Nurses	27(21.09)	101 (78.91)	128(100)
Technicians	2 (28.57)	5(71.43)	7(100)
Total	89 (36.48)	155(63.52)	244 (100)

Nurses and technicians were clubbed for the analysis, χ^2^ = 29.69 df = 2, *P* = 0.00000036, HCW - Health care worker. Figures in parentheses are in percentage

#### Details of lab investigations:

Immediate post-exposure ELISA for HIV was done in all those who underwent lab investigations. However, out of the total 89 HCWs who underwent ELISA post-exposure, 27 did not undergo ELISA at 12 weeks or later. Only 19 HCWs underwent test for HBsAG, as most (211), i.e., 86% had taken Hepatitis B vaccination. None of the HCWs tested HIV positive by ELISA. Four of the nurses who underwent testing for HBsAg were positive for Hepatitis B. However, there was insufficient evidence to link their Hepatitis B positive status to occupational exposure.

#### Number of HCWs taking PEP category-wise:

This is shown in Table 5. Only 21.31% of the HCWs exposed to blood and body fluids took PEP for HIV (though the same was indicated in about 50% of the cases of exposure). The proportion of residents and interns (20% and 33.9%, respectively) who took PEP was greater than those of nurses (14.06%).

**Table 5:** Number of HCWs taking PEP category-wise

Category of HCW	Took PEP (%)	Total (%)
Residents	10(20)	50(100)
Interns	20 (33.90)	59(100)
Nurses	18(14.06)	128(100)
Technicians	4 (57.14)	7(100)
Total	52(21.31)	244(100)

Nurses and technicians were clubbed for the analysis, χ^2^ = 7.65, df = 2, *P* = 0.02

## Discussion

Self-reported occupational exposure to blood and body fluids in the preceding 12 months was fairly high, ranging from the lowest incidence of 21% among residents to more than 39% among the nurses. The present study showed the highest incidence of occupational exposure among nurses. It has been reported that nurses experience the majority of needle-stick injuries in the world including half of the exposures that occur in the US(4,5) and 70% of exposures occurring in Canada.(6) Among junior doctors, interns had a higher incidence of exposure as compared to residents. This may be due to their inexperience in practical procedures. Clarke *et al*.,(7) in their study, found that the probability of ever having a needle-stick injury was inversely related to years of experience.

Majority of accidental exposures to blood and body fluids was due to needle-stick injuries and most of them were percutaneous. In developing countries, where the prevalence of HIV-infected patients is the highest in the world, the number of needle-stick injuries is also the highest.(8) In some regions of Africa and Asia, close to half of all Hepatitis B and C infections among HCWs are attributable to contaminated sharps. Factors surrounding the circumstances of the needle-stick injury, when combined, can increase the risk of HIV infection to 1 in 20 (or 5% risk). These factors include a deep injury, visible blood on the device, high viral titre status of the patient such as in newly infected patients or those in a terminal state, and the device being used to access an artery or a vein.(9)

Unreported needle-stick and sharp injuries are a serious problem and prevent injured HCWs from receiving PEP against HIV, which is shown to be 80% effective against HIV infection.(8) According to researchers, 40%-70% of all needle-stick injuries are unreported.(8) Without documentation, of the injury, the affected HCW is unlikely to receive worker's compensation benefits if later becoming infected with the HIV or other blood-borne pathogens.

Less than a quarter of the exposed HCWs took a course of PEP against HIV, though it appears that the same was indicated in about half of the affected HCWs. This low rate of PEP was due to under-reporting to concerned hospital authorities. Clarke *et al*.,(7) in their study, found that only 29% of exposed respondents reported the incident. Reasons for not reporting included: the source thought it to be non-infectious, insignificant exposure, too little time to report, already immunized for Hepatitis B, the outcome remaining unchanged by reporting, the exposure was not an emergency and not knowing how to report an exposure. These reasons accounted for 83% of the reasons given for not reporting.

The United States National Surveillance System for Health Care Workers (NaSH) identified six devices that are responsible for the majority of needle-stick and other sharp-related injuries. These are hypodermic needles (32%), suture needles (19%), winged steel needles (butterfly) (12%), scalpel blades, IV catheter stylets (96%) and phlebotomy needles (3%).(4) Percutaneous or needle-stick injuries contaminated with blood or body fluids pose the highest risk and cause the most common exposures among HCWs.(4) These blood-filled devices account for 59% of all NaSH reported and 90% of the HIV seroconversion documented by CDC.(4) The most common circumstances that cause injuries in NaSH hospitals involve hollow bore needles, which are the most risky because these needles can be filled with blood. Situations of injury include the following: manipulating the patient (26%), disposal (23%), collision with worker or sharps (10%), during clean-up (10%), accessing IV lines (6%) and re-capping needles (6%).(4)

The use of data collected about the nature of occupational exposures, needle-stick injuries and near-misses helps guide prevention at the unit or institutional level and helps make recommendations for new practices and devices for prevention and re-occurrence of injuries. In 2004, the CDC published a web-based resource: Workbook for Designing, Implementing and Evaluating a Sharps Injury Prevention Program.(4) The workbook describes the use of Root Cause Analysis, a process for identifying causal factors to use in needle-stick injury prevention, and suggests that the institution's needle-stick prevention committee ask key questions (What happened? How did it happen? Why did it happen? What can be done to prevent it from happening in the future?) to get at the “root” of situations resulting in injuries, and thus identifying areas for change.(4) By identifying why and how injuries occur in specific settings, interventions can be easily recognized and prioritized. Reporting injuries and documenting all blood-borne exposures are essential for having the evidence to analyze for prevention.

A number of studies have explored needle-stick injuries among HCWs.(10–20) Because of the differences between studies, it is not possible to quantitatively synthesize their results; nonetheless, some common themes emerge, such as - needle-stick injuries are common; needle-stick injuries are often under-reported and when levels of reporting have been examined, it is common for only a small proportion to be reported; and knowledge about needle-stick injuries and possible infection from blood-borne pathogens is often low and risks under-estimated.

The present study also reiterates the above themes, particularly the first two, namely that needle-stick injuries are a fairly common occurrence among HCWs and secondly that they are grossly under-reported. The incidence of exposure to blood and body fluids in the present study was measured by self-reporting on the part of the HCW. This may have led to inaccuracies in the true incidence due to recall bias.

To have a proper database on these injuries, developing countries should also develop surveillance systems for needle-stick injuries among HCWs. Legal measures are also indicated to address compensation for HCWs who contact blood-borne pathogens as an occupational hazard. All these would require proper notification, documentation and education of HCWs.
